# Decoding Fujian’s cervical HPV landscape: unmasking dominance of non-16/18 HR-HPV and tailoring prevention strategies at a large scale

**DOI:** 10.3389/fpubh.2024.1357073

**Published:** 2024-06-06

**Authors:** Yulong Zhang, Haibo Li, Qianru You, Yusha Chen, Ziyan Zhao, Jiancui Chen, Yanzhao Su, Xiangqin Zheng, Huan Yi, Jianrong Song

**Affiliations:** ^1^Department of Gynecology, Fujian Province Key Clinical Specialty for Gynecology, National Key Gynecology Clinical Specialty Building Institution, Fujian Maternity and Child Health Hospital College of Clinical Medical for Obstetrics & Gynecology and Pediatrics, Fujian Medical University, Fuzhou, China; ^2^Division of Birth Cohort Study, Fujian Maternity and Child Health Hospital, College of Clinical Medicine for Obstetrics & Gynecology and Pediatrics, Fujian Medical University, Fuzhou, China; ^3^Cervical Disease Diagnosis and Treatment Health Center, Fujian Maternity and Child Health Hospital College of Clinical Medical for Obstetrics & Gynecology and Pediatrics, Fujian Medical University, Fuzhou, China; ^4^Department of Integrative Biology, University of California-Berkeley, Berkeley, CA, United States

**Keywords:** cervical cancer, human papillomavirus, genotype, prevalence, prevention

## Abstract

**Background:**

Persistent HR-HPV causes cervical cancer, exhibiting geographic variance. Europe/Americas have higher HPV16/18 rates, while Asia/Africa predominantly have non-16/18 HR-HPV. This study in Fujian, Asia, explores non-16/18 HR-HPV infections, assessing their epidemiology and cervical lesion association for targeted prevention.

**Methods:**

A total of 101,621 women undergoing HPV screening at a hospital in Fujian Province from 2013 to 2019 were included. HPV genotyping was performed. A subset of 11,666 HPV-positive women with available histopathology results were analyzed to characterize HPV genotype distribution across cervical diagnoses.

**Results:**

In 101,621 samples, 24.5% tested positive for HPV. Among these samples, 17.3% exhibited single infections, while 7.2% showed evidence of multiple infections. The predominant non-16/18 high-risk HPV types identified were HPV 52, 58, 53, 51, and 81. Single HPV infections accounted for 64.1% of all HPV-positive cases, with 71.4% of these being non-16/18 high-risk HPV infections. Age-related variations were observed in 11,666 HPV-positive patients with pathological results. Cancer patients were older. In the cancer group, HPV52 (21.8%) and HPV58 (18.6%) were the predominant types, followed by HPV33, HPV31, and HPV53. Compared to single HPV16/18 infection, non-16/18 HPV predominated in LSIL. Adjusted odds ratios (OR) for LSIL were elevated: multiple HPV16/18 (OR 2.18), multiple non-16/18 HR-HPV (OR 2.53), and multiple LR-HPV (OR 2.38). Notably, solitary HPV16/18 conferred higher odds for HSIL and cancer.

**Conclusion:**

Our large-scale analysis in Fujian Province highlights HPV 52, 58, 53, 51, and 81 as predominant non-16/18 HR-HPV types. Multiple HPV poses increased LSIL risks, while solitary HPV16/18 elevates HSIL and cancer odds. These findings stress tailored cervical cancer prevention, highlighting specific HPV impacts on lesion severity and guiding region-specific strategies for optimal screening in Asia, emphasizing ongoing surveillance in the vaccination era.

## 1 Introduction

Cervical cancer, a significant global public health burden, exacts a heavy toll in terms of morbidity and mortality (1). The 2020 World Cancer Report underscores this by highlighting that cervical cancer comprises 6.5% of all newly diagnosed cancers in women worldwide and ranks as the fourth leading cause of female cancer deaths (2). In China alone, nearly 20% of the global burden is shouldered, with approximately 98,900 new cases reported annually and an alarming 30,500 women succumbing to the disease each year (3).

Although the exact causes are multifactorial, persistent infection with certain strains of human papillomavirus (HPV) has been identified as the primary driving force behind the development of cervical cancer and precancerous cervical lesions (3). This viral link provides a potential avenue for prevention and control strategies. The pivotal role of persistent infection with high-risk human papillomavirus (HR-HPV) genotypes in the pathogenesis of cervical cancer and its precursor lesions cannot be overstated (3). Over 90% of cervical cancers can be attributed to HR-HPV, making it the primary risk factor (4, 5). HPV16 and HPV18 dominate globally as the most prevalent HR-HPV types detected in cervical cancers (6). However, in recent years, cervical tumors caused by other HPV types have been gradually increasing, warranting sufficient attention (7).

Given the established causal relationship between HPV and cervical cancer, the development of prophylactic HPV vaccines has emerged as a promising preventive strategy. Various HPV vaccines, including bivalent, quadrivalent, and nine-valent formulations, have been developed and introduced into vaccination programs worldwide. The nine-valent vaccine offers the broadest protection against HPV6/11/16/18/31/33/45/52/58, with an estimated potential effectiveness against cervical cancer in China of 75.4% (8). The bivalent HPV16/18 vaccine could prevent 55.4% of cases (9). However, these vaccines do not provide universal protection, as they target specific genotypes.

While current vaccines target the globally prevalent HPV16/18 strains, regional variations exist, with non-16/18 HR-HPV types being more dominant in certain regions like Asia. Therefore, assessing the oncogenicity of non-vaccine HR-HPV types remains crucial, as they may still pose cancer risks in vaccinated groups (10). This study aims to characterize the epidemiology of non-HPV 16/18 high-risk infections in Fujian Province and their association with cervical lesions, considering their dominance in Asia over global prototypical strains. Elucidating prevalent regional genotypes in China and their cancer linkage, alongside prevailing vaccine usage, provides critical insights for optimized, tailored screening and the development of next-generation prophylactics expanding genotype coverage and effectiveness to ultimately enhance cervical cancer prevention in this population.

## 2 Methods

### 2.1 Study design and participants

**Figure 1:**
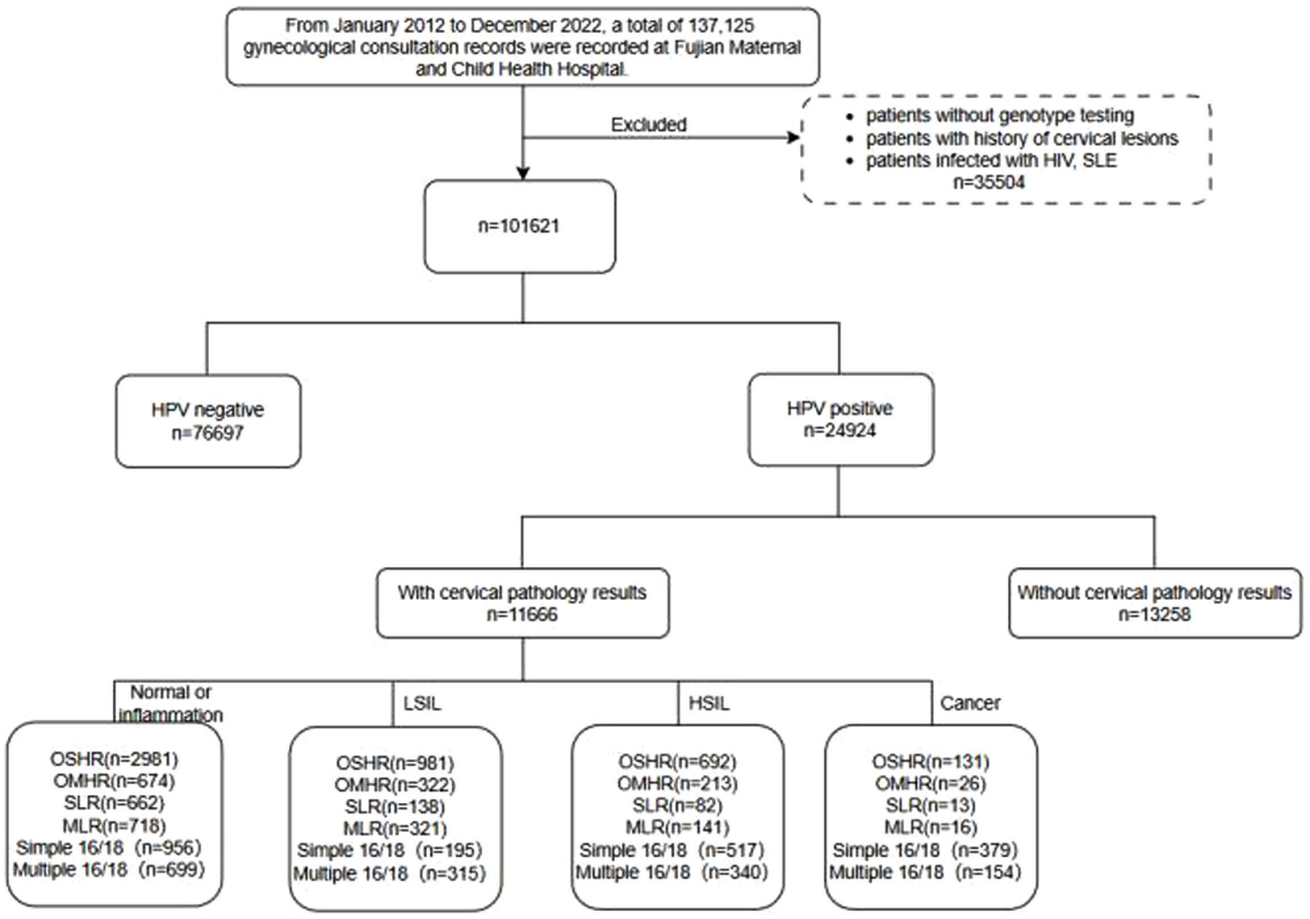
The flowchart of HPV infection patients. LSIL, low-grade squamous intraepithelial lesion; HSIL, high-grade squamous intraepithelial lesion; OSHR, non-16/18 high-risk HPV infection; OMHR, multiple non-16/18 high-risk HPV infection; SLR, low-risk HPV infection; MLR, multiple low-risk HPV infection; SLE, systemic lupus erythematosus.

This retrospective cohort study was conducted at Fujian Maternity and Child Health Hospital in Fuzhou, China, covering the period from January 2012 to December 2022. A total of 137,125 clinic records were initially screened. Participants who were under 18 years old, lacked HPV typing, had a history of cervical diseases, had immune defects, or were on immunosuppressive drugs (e.g., HIV or SLE, systemic lupus erythematosus), as well as those with missing or invalid data, were excluded from the study. After the exclusion criteria were applied, 24,924 patients infected with HPV were included for further screening. From this group, patients lacking relevant pathological examinations were excluded, leaving 11,666 patients divided into four groups: normal or inflammation, LSIL, HSIL, and cancer. The study flow chart is depicted in Figure 1.

Ethical approval for this study was obtained from the Research Ethics Committee of Fujian Maternity and Child Health Hospital (2023KY038). To protect patient privacy, all data related to individual identification were removed and remained anonymous throughout the analysis. The study involved retrospective collection of patient information, and an informed consent exemption was obtained.

### 2.2 HPV genotype and cervical pathology

PCR-RDB HPV genotyping (Yaneng Biotech) was conducted to identify 18 types of high-risk HPV (HR-HPV) (16/18/31/33/35/39/45/51/52/53/56/58/59/66/68/73/82/83) and 5 types of low-risk HPV (LR-HPV) (6/11/42/43/81) among the study participants. Based on their HPV infection statuses, the patients were categorized into six groups: 16/18 simplex infection group, 16/18 multiple-infection group, low-risk simplex infection group, non-16/18 high-risk simplex infection group, low-risk multiple-infection group, and non-16/18 high-risk multiple-infection group.

Pathological findings were determined from cervical tissue samples obtained after standardized colposcopy. These samples were diagnosed by a consistent histopathologist who remained blinded to the participants’ HPV statuses throughout the study.

### 2.3 Statistical analysis

All patients were stratified by age into the following categories: < 25, 25–29, 30–34, 35–39, 40–44, 45–49, 50–54, 55–59, 60–64, and ≥ 65 years. Descriptive statistics for continuous variables were presented as mean ± standard deviation and analyzed using one-way analysis of variance (ANOVA). Categorical variables were presented as percentages and analyzed using the chi-square test (or Fisher test).

Additionally, multivariate multinomial logistic regressions were conducted to determine the relationships between the various HPV infection types and final pathological diagnoses, controlling for potential confounding factors such as age and year. The statistical analysis was performed using R software and its software packages (https://www.r-project.org, Version 4.2.2). All *p*-values were two-tailed, and a significance level of *p* < 0.05 was considered statistically significant.

## 3 Results

### 3.1 General distribution of HPV infections

**Figure 2:**
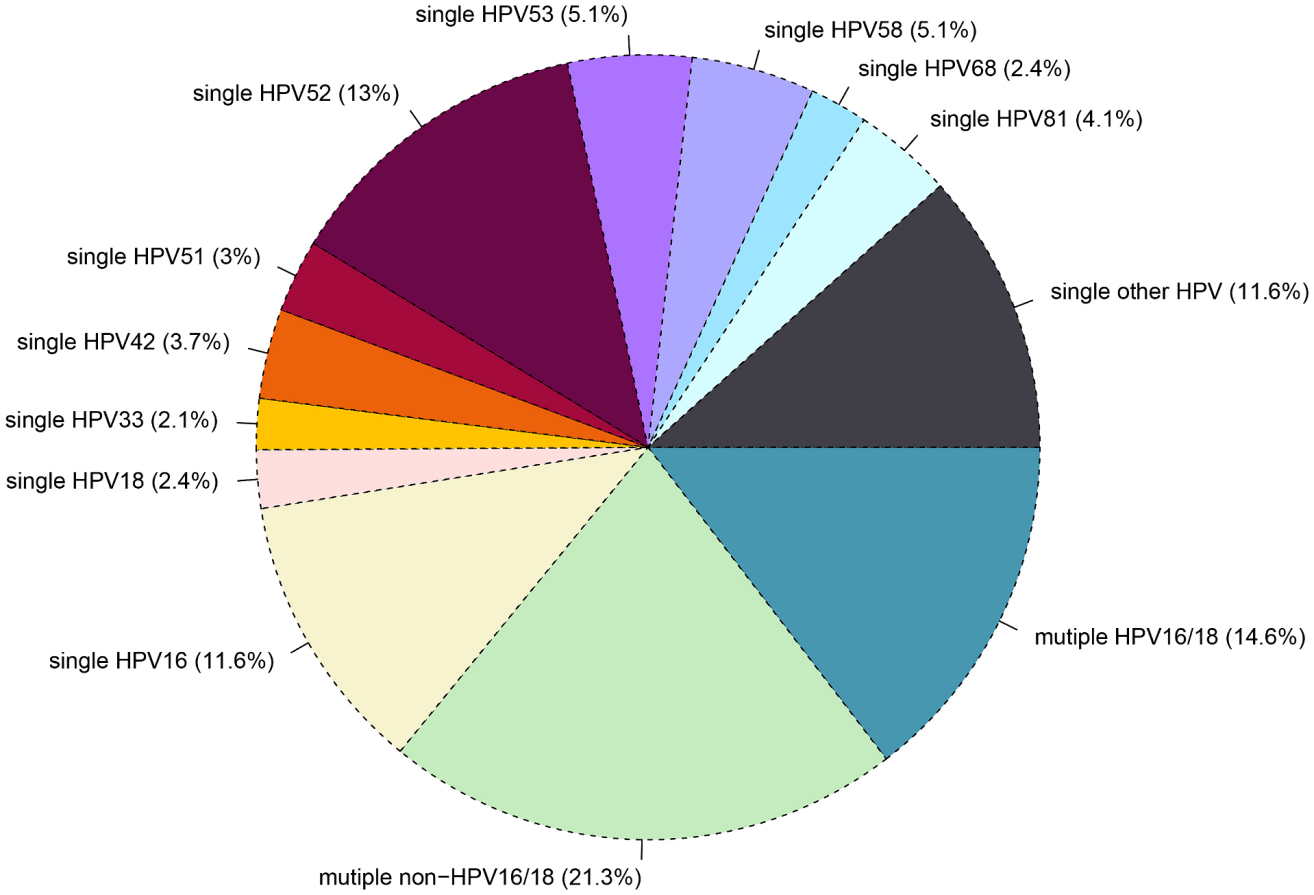
Distribution of single and multiple infections among different HR-HPV subtypes.

Out of the 101,621 samples analyzed, 24,924 (24.5%) tested positive for HPV. Among these samples. The patients were categorized into single and multiple infection groups based on the number of HPV types detected. The overall rate of single HPV infection was 17.3%, with 2.8% exhibiting a single infection with HPV16/18, 11.3% showing other high-risk HPV infections (OSHR), and 3.2% demonstrating single low-risk HPV infections (SLR). Multiple HPV infections occurred in 7.2% of samples, with 2.2% having multiple infections with HPV16/18, 2.4% showing other multiple high-risk HPV infections (OMHR), and 2.6% exhibiting multiple low-risk HPV infections (MLR), as shown in Table 1. As depicted in Figure 2, the top five genotypes for single HPV infection were HPV 52 (13%), HPV 16 (11.6%), HPV 53 (5.1%), HPV 58 (5.1%), HPV 81 (4.1%), and HPV 42 (3.7%), with their respective infection rates mentioned in parentheses. The overall non-16/18 HPV infection rate was 71.4%, indicating that the majority of infections were attributed to HPV types other than 16 and 18. Furthermore, the rate of multiple non-16/18 HPV infections (21.3%) exceeded that of multiple 16/18 infections (14.6%), underscoring the significance of non-16/18 HPV types in multiple infections.

**Table 1:** HPV infection of different subtypes in different age groups.

Variables	Total (*n* = 101,621)	<25 (*n* = 5,766)	25–29 (*n* = 16,661)	30–34 (*n* = 20,033)	35–39 (*n* = 15,951)	40–44 (*n* = 14,610)	45–49 (*n* = 12,725)	50–54 (*n* = 7,872)	55–59 (*n* = 3,984)	60–64 (*n* = 2096)	≥65 (*n* = 1923)
HPVgenotype, *n* (%)											
Negative	76,697 (75.5)	3,984 (69.1)	13,040 (78.3)	15,842 (79.1)	12,417 (77.8)	11,117 (76.1)	9,546 (75)	5,666 (72)	2,475 (62.1)	1,259 (60.1)	1,351 (70.3)
hpv16/18single	2,890 (2.8)	166 (2.9)	384 (2.3)	465 (2.3)	420 (2.6)	455 (3.1)	416 (3.3)	272 (3.5)	169 (4.2)	96 (4.6)	47 (2.4)
OSHR	11,480 (11.3)	663 (11.5)	1,643 (9.9)	2039 (10.2)	1751 (11)	1733 (11.9)	1,506 (11.8)	1,012 (12.9)	613 (15.4)	316 (15.1)	204 (10.6)
SLR	3,241 (3.2)	219 (3.8)	528 (3.2)	532 (2.7)	446 (2.8)	486 (3.3)	443 (3.5)	298 (3.8)	171 (4.3)	61 (2.9)	57 (3)
Multiple 16/18	2,195 (2.2)	254 (4.4)	313 (1.9)	356 (1.8)	265 (1.7)	234 (1.6)	244 (1.9)	178 (2.3)	170 (4.3)	105 (5)	76 (4)
OMHR	2,463 (2.4)	207 (3.6)	351 (2.1)	409 (2)	332 (2.1)	318 (2.2)	266 (2.1)	205 (2.6)	175 (4.4)	119 (5.7)	81 (4.2)
MLR	2,655 (2.6)	273 (4.7)	402 (2.4)	390 (1.9)	320 (2)	267 (1.8)	304 (2.4)	241 (3.1)	211 (5.3)	140 (6.7)	107 (5.6)

OSHR, non-16/18 high-risk HPV infection; OMHR, multiple non-16/18 high-risk HPV infection; SLR, low-risk HPV infection; MLR, multiple low-risk HPV infection.

### 3.2 Distribution of HPV subtypes at different ages and years

Age-related variations were observed in Table 1. The HPV-negative group peaked at 78.3% in those aged 25–29 years and gradually declined with increasing age, reaching a nadir of 62.1% in the 55–59 year age group before slightly rebounding in those ≥65 years (70.3%). HPV 16/18 single infections exhibited a bimodal pattern, reaching peaks of 2.9% in individuals <25 years and 4.6% in the 60–64 year age group. Other high-risk (OSHR) genotypes were elevated at 11.5% in individuals <25 years, remained relatively stable from 25–49 years (9.9–11.9%), then sharply increased after age 55, peaking at 15.4% in the 55–59 year age group. Low-risk (SLR) types showed minor peaks in individuals <25 years (3.8%) and the 55–59 year age group (4.3%). Multiple HPV 16/18 infections were highest in individuals <25 years (4.4%), low from 25–49 years (1.6–1.9%), then rose after age 50. Other multiple high-risk (OMHR) infections were 3.6% in individuals <25 years, 2.1–2.2% from 25–49 years, increasing after age 50 to peak at 5.7% in the 60–64 year age group. Multiple low-risk (MLR) infections peaked at 4.7% in individuals <25 years, were 1.8–2.4% from 25–49 years, then increased markedly after age 50, reaching 6.7% in the 60–64 year age group.

These age-related variations suggest dynamic changes in HPV genotype distribution across different age groups. In younger individuals, particularly those <25 years old, there appears to be a higher prevalence of HPV 16/18 single infections and multiple HPV infections involving HPV 16/18. However, as age increases, there is a notable shift towards other high-risk HPV genotypes (OSHR), with a marked increase in prevalence after age 55, peaking in the 55–59 year age group. This transition may reflect changes in sexual behavior, immune response, or other factors influencing HPV acquisition, persistence, and clearance over the course of an individual’s lifespan.

**Figure 3:**
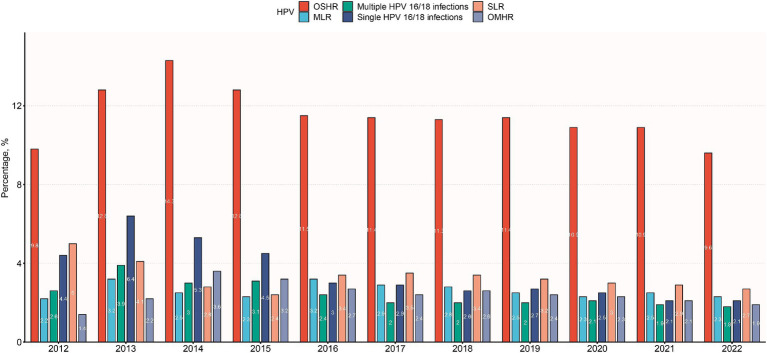
Distribution of HPV infection subtypes among different years. OSHR, non-16/18 high-risk HPV infection; OMHR, multiple non-16/18 high-risk HPV infection; SLR, low-risk HPV infection; MLR, multiple low-risk HPV infection.

The trend depicted in Figure 3 illustrates varying HPV infection rates over time. Single HPV 16/18 infections peaked in 2013, gradually declining to 2.1% by 2022. Similarly, multiple HPV 16/18 infections peaked in 2013, decreasing to 1.8% by 2022. Other high-risk (OSHR) infections peaked in 2014, remaining relatively stable between 9.6–12.8%. Other multiple high-risk (OMHR) infections peaked in 2014, declining to 1.9% by 2022. Low-risk (SLR) infections peaked in 2012, decreasing to 2.7% by 2022. Multiple low-risk (MLR) infections had minor peaks in 2013 and 2016–2017, remaining stable at 2.2–2.8%. Overall, HPV infections peaked around 2013–2014 before declining, highlighting vaccine effectiveness. Persistent high-risk strains and age-related variations emphasize the need for ongoing surveillance and targeted interventions.

### 3.3 Characterizing cervical lesions in HPV-infected patients undergoing pathological examination

**Figure 4:**
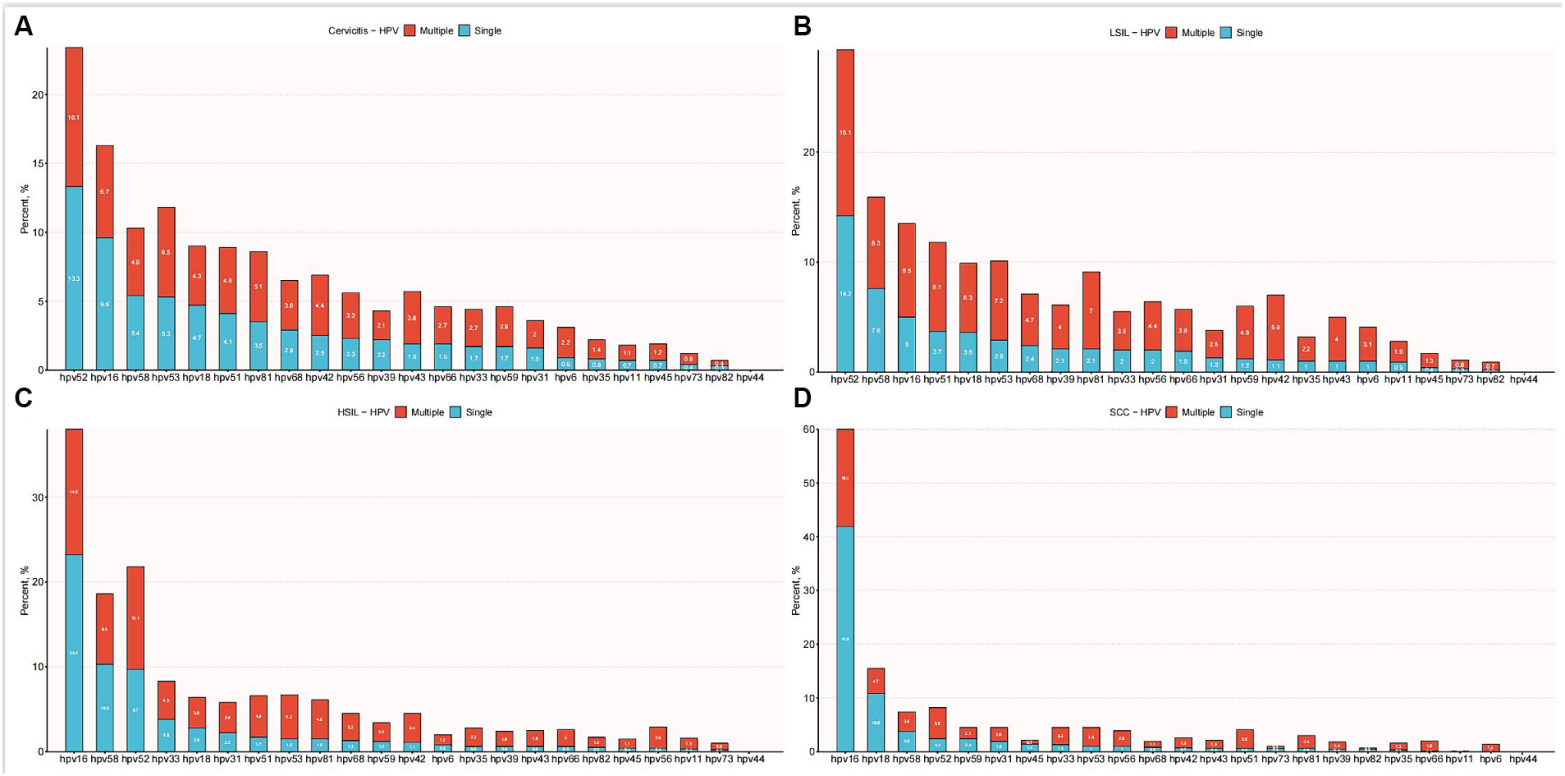
Distribution of single and multiple infections among different HPV subtypes. **(A)** Distribution of multiple and single HPV genotype infections in patients with cervicitis. **(B)** Distribution of multiple and single HPV genotype infections in patients with LSIL. **(C)** Distribution of multiple and single HPV genotype infections in patients with HSIL. **(D)** Distribution of multiple and single HPV genotype infections in patients with SCC.

Table 2 illustrates the frequencies of non-16/18 high-risk HPV types and their distribution across different grades of cervical lesions. This large-scale analysis involved 11,666 HPV-positive patients with cervical pathology. The results revealed a significant association between age distribution and pathology type (*p* < 0.001). Patients with cervical cancer were older, with a mean age of 49 years, compared to those with other pathologies, who ranged from 38 to 42 years of age. The analysis reveals the prevalence of various non-16/18 high-risk HPV types among different cervical lesion groups. In the cancer group, HPV52 (8.2%) and HPV58 (7.4%) were the predominant types, followed by HPV33, HPV31, and HPV53. In the High-grade Squamous Intraepithelial Lesion (HSIL) group, HPV52 remained prevalent at 21.8%, along with significant proportions of HPV58, HPV33, HPV31, and HPV53. Similarly, in the Low-grade Squamous Intraepithelial Lesion (LSIL) group, HPV52 was the most common type at 29.3%, with HPV58, HPV33, HPV31, and HPV53 also exhibiting notable frequencies. The distribution of single and multiple infections by gene typing is illustrated in Figure 4. These findings underscore the importance of considering the distribution and potential contributions of non-16/18 high-risk HPV types in the development and progression of cervical lesions across different patient populations.

**Table 2:** Distribution of each human papillomavirus genotypes according to cervical pathology results among the 11,666 HPV infected patients, *n* (%).

Variables	Total (*n* = 11,666)	Cancer (*n* = 719)	HSIL (*n* = 1985)	LSIL (*n* = 2,272)	Normal or cervicitis (*n* = 6,690)	*p*
HPV age, Mean ± SD	41.9 ± 11.3	49.5 ± 10.0	42.5 ± 11.3	38.0 ± 10.7	42.2 ± 11.1	< 0.001
hpv16, *n* (%)	2,583 (22.1)	431 (59.9)	754 (38)	307 (13.5)	1,091 (16.3)	< 0.001
hpv18, *n* (%)	1,068 (9.2)	112 (15.6)	127 (6.4)	225 (9.9)	604 (9)	< 0.001
hpv31, *n* (%)	478 (4.1)	33 (4.6)	116 (5.8)	86 (3.8)	243 (3.6)	< 0.001
hpv33, *n* (%)	613 (5.3)	32 (4.5)	165 (8.3)	125 (5.5)	291 (4.3)	< 0.001
hpv35, *n* (%)	291 (2.5)	11 (1.5)	56 (2.8)	74 (3.3)	150 (2.2)	< 0.001
hpv39, *n* (%)	484 (4.1)	13 (1.8)	46 (2.3)	138 (6.1)	287 (4.3)	< 0.001
hpv45, *n* (%)	209 (1.8)	15 (2.1)	29 (1.5)	39 (1.7)	126 (1.9)	< 0.001
hpv51, *n* (%)	1,018 (8.7)	29 (4)	130 (6.5)	268 (11.8)	591 (8.8)	< 0.001
hpv52, *n* (%)	2,727 (23.4)	59 (8.2)	433 (21.8)	666 (29.3)	1,569 (23.5)	< 0.001
hpv53, *n* (%)	1,184 (10.1)	32 (4.5)	133 (6.7)	228 (10)	791 (11.8)	< 0.001
hpv56, *n* (%)	611 (5.2)	28 (3.9)	58 (2.9)	147 (6.5)	378 (5.7)	< 0.001
hpv58, *n* (%)	1,474 (12.6)	53 (7.4)	370 (18.6)	361 (15.9)	690 (10.3)	< 0.001
hpv59, *n* (%)	539 (4.6)	32 (4.5)	67 (3.4)	137 (6)	303 (4.5)	< 0.001
hpv66, *n* (%)	503 (4.3)	15 (2.1)	50 (2.5)	130 (5.7)	308 (4.6)	< 0.001
hpv68, *n* (%)	697 (6.0)	14 (1.9)	90 (4.5)	162 (7.1)	431 (6.4)	< 0.001
hpv73, *n* (%)	130 (1.1)	7 (1)	19 (1)	26 (1.1)	78 (1.2)	< 0.001
hpv82, *n* (%)	107 (0.9)	5 (0.7)	32 (1.6)	20 (0.9)	50 (0.7)	< 0.001
hpv42, *n* (%)	729 (6.2)	19 (2.6)	88 (4.4)	160 (7)	462 (6.9)	< 0.001
hpv43, *n* (%)	554 (4.7)	15 (2.1)	48 (2.4)	112 (4.9)	379 (5.7)	< 0.001
hpv44, *n* (%)	3 (0.0)	0 (0)	0 (0)	0 (0)	3 (0)	< 0.001
hpv11, *n* (%)	213 (1.8)	1 (0.1)	31 (1.6)	63 (2.8)	118 (1.8)	< 0.001
hpv6, *n* (%)	347 (3.0)	10 (1.4)	39 (2)	92 (4)	206 (3.1)	< 0.001
hpv81, *n* (%)	926 (7.9)	21 (2.9)	120 (6)	207 (9.1)	578 (8.6)	< 0.001
hpv83, *n* (%)	110 (0.9)	4 (0.6)	13 (0.7)	19 (0.8)	74 (1.1)	< 0.001

### 3.4 Impact of HPV infection subtypes on cervical pathology

To gain further insights into the correlation between HPV types and the severity of cervical lesions, additional analyses were conducted by the researchers, adjusting for age and year (Table 3). Compared to single HPV16/18 infection, the following HPV types showed higher odds of association with LSIL: multiple HPV16/18 infections (OR 2.18, 95% CI 1.77–2.68), non-16/18 single high-risk HPV (OSHR) (OR 1.71, 95% CI 1.44–2.04), non-16/18 multiple high-risk HPV (OMHR) (OR 2.53, 95% CI 2.05–3.12), single low-risk HPV (SLR) (OR 1.08, 95% CI 0.85–1.38), and multiple low-risk HPV (MLR) (OR 2.38, 95% CI 1.93–2.93). However, solitary HPV16/18 infections conferred higher odds of association with HSIL and cervical cancer compared to other HPV types. These findings suggest that while multiple infections and non-16/18 high-risk HPV types increase the risk of LSIL, HPV16 and HPV18 play a more prominent role in the progression to more severe cervical lesions and cervical cancer. Understanding these associations informs targeted prevention strategies and underscores the importance of HPV vaccination and vigilant screening programs for cervical cancer prevention.

**Table 3:** Multivariate logistic regression analysis of HPV infection types and final diagnoses.

Variable	No of total	normal or cervicitis, *n* (%)	LSIL, *n* (%)	HSIL, *n* (%)	Cancer, *n* (%)	LSIL vs. normal or cervicitis		HSIL vs. normal or cervicitis		Cancer vs. normal or cervicitis	
						OR (95%CI)	*p* value	OR (95%CI)	*p* value	OR (95%CI)	*p* value
Simple hpv16/18	2,047	956 (46.7)	195 (9.5)	517 (25.3)	379 (18.5)	1 (Ref)		1 (Ref)		1 (Ref)	
Multiple hpv 16/18	1,508	699 (46.4)	315 (20.9)	340 (22.5)	154 (10.2)	2.21 (1.8 ~ 2.71)	<0.001	0.9 (0.76 ~ 1.06)	0.216	0.56 (0.45 ~ 0.69)	<0.001
OSHR	4,785	2,981 (62.3)	981 (20.5)	692 (14.5)	131 (2.7)	1.61 (1.36 ~ 1.91)	<0.001	0.43 (0.37 ~ 0.49)	<0.001	0.11 (0.09 ~ 0.14)	<0.001
OMHR	1,235	674 (54.6)	322 (26.1)	213 (17.2)	26 (2.1)	2.34 (1.91 ~ 2.87)	<0.001	0.58 (0.48 ~ 0.7)	<0.001	0.1 (0.06 ~ 0.15)	<0.001
SLR	895	662 (74)	138 (15.4)	82 (9.2)	13 (1.5)	1.02 (0.8 ~ 1.3)	0.859	0.23 (0.18 ~ 0.3)	<0.001	0.05 (0.03 ~ 0.09)	<0.001
MLR	1,196	718 (60)	321 (26.8)	141 (11.8)	16 (1.3)	2.19 (1.79 ~ 2.68)	<0.001	0.36 (0.29 ~ 0.45)	<0.001	0.06 (0.03 ~ 0.09)	<0.001

Model adjusted age and years.

OSHR, non-16/18 high-risk HPV infection; SLR:low-risk HPV infectio; OMHR:multiple non-16/18 high-risk HPV infection; MLR, multiple low-risk HPV infection.

## 4 Discussion

Previous studies have found that HPV16 and 18 are responsible for approximately 70% of cervical cancers worldwide (11). The introduction of HPV vaccines holds promise in preventing HPV16/18-associated cancers, supported by the safety and efficacy of current vaccines (12). However, in the post-vaccine era, understanding HPV genotype distribution, non-16/18 epidemiology, and associated cancer risks is pivotal for optimizing vaccine development. This knowledge also guides screening and management of cervical lesions, particularly in less explored regions like Fujian Province, China.

Against this backdrop of limited vaccine coverage, assessing the oncogenicity of non-vaccine high-risk HPV types remains crucial, as they may still pose cancer risks in vaccinated groups. Our study findings reveal a widespread prevalence of non-16/18 HPV types, accounting for substantial proportions in cervical lesions across Fujian Province. This underscores the significance of these oft-neglected genotypes in cervical carcinogenesis within this region. Specifically, our data highlight the prominent roles of HPV58, HPV33, and HPV52 among the high-risk non-16/18 types implicated in precancerous cervical intraepithelial neoplasia and invasive cervical cancer cases. These results underscore the need for comprehensive genotyping to delineate region-specific HPV profiles and cancer associations for informing tailored prevention and management strategies. Our findings revealed an HPV infection rate of 24.5%, surpassing the overall Asian rate (8.3%) (13) but falling below a prior estimate for China (47.3%) (14). The prevalent types, including HPV52, 16, 58, 51, and 53, mirrored China’s dominant strains (14). Geographic variations in HPV prevalence indicate the influence of diverse factors, emphasizing the need for region-specific studies (15).

Building upon these insights into the regional HPV landscape, we identified 17.3% single HPV and 7.2% multiple HPV infections. Notably, multiple non-16/18 infections exceeded multiple 16/18 infections. While multiple HPV infection may not independently increase cervical cancer risk (15), some studies associate it with cancer-related genotypes and prolonged infections (16). Our findings therefore warrant further research on potential synergistic or competitive effects of multiple HPVs in cervical carcinogenesis.

Moreover, our investigation into age distribution uncovered a notable bimodal pattern in infection rates. This bimodal trend aligns with findings reported elsewhere (17). Proposed explanations posit that heightened sexual activity and engagement with new/multiple partners contribute to the peak in younger women, alongside relatively immature anti-HPV immunity. Conversely, the second peak in older women may be attributed to hormonal changes and potential reactivation in individuals seeking care for symptoms of prior infection (17, 18). Importantly, prior research suggests that this bimodal distribution may be influenced by lower levels of urbanization and education (19). Furthermore, understanding these trends can guide targeted interventions to mitigate HPV transmission risks. These age-specific infection rates lay the groundwork for our subsequent investigations, shedding light on the dynamic interplay between HPV prevalence and demographic factors.

Additionally, the infection rate of non-16/18 multiple types of HPV peaked in 2014 at 3.6%, gradually decreasing annually thereafter. By 2022, it had reached 1.9%, indicating potential improvements in detection methods accompanying economic development. However, despite this stabilization, the overall infection rate remains higher than the global average (20). Therefore, ongoing initiatives to enhance screening accessibility and vaccine coverage remain paramount. Moreover, the escalating proportion of non-16/18 HR-HPV and low-risk HPV underscores the importance of transmission prevention and asymptomatic infection management. Understanding these temporal trends in HPV prevalence highlights the significance of continued research into age-specific distribution, guiding more effective strategies for HPV control and prevention.

Furthermore, a deeper analysis of HPV genotype distribution’s impact on pathological outcomes is essential. Persistent infection with high-risk HPV strains is recognized to increase the risk of developing precancerous and cancerous lesions (21–23). In benign infections, HPV typically remains in an episomal free form, but integration into the host DNA can occur, resulting in the overexpression of viral oncogenes E6 and E7. This dysregulated gene expression promotes cellular proliferation, genomic instability, and lesion progression (24, 25). However, despite this understanding, the precise interactions between specific HPV types and their roles in lesion advancement remain unclear and necessitate further investigation (26). Elucidating these interactions is crucial for better understanding HPV-related disease pathogenesis and developing more targeted interventions for prevention and treatment.

Therefore, studying the distribution characteristics of HPV types in different cervical lesions is paramount. In a cohort of 11,666 HPV-positive patients, significant variations in HPV type distribution were noted across different pathological outcomes (*p* < 0.001). Specifically, non-16/18 single high-risk HPV (HR-HPV) types were prevalent in low-grade squamous intraepithelial lesions (LSIL) and high-grade squamous intraepithelial lesions (HSIL), respectively. In contrast, cancer patients exhibited the highest rate of solitary HPV16/18 infections. Moreover, certain HPV types were found to be more strongly associated with specific disease states, with other HPV types being more common in LSIL, while solitary HPV16/18 infections were correlated with HSIL and cancer. LSIL and HSIL showed non-16/18 single infections predominating, while solitary HPV16/18 was highest in cancer. An inverse association between multiple HPV and lesion severity has been reported (27). In summary, HPV16/18 predominates in cancer, while non-16/18 types prevail in LSIL and HSIL. Targeting non-16/18 genotypes like 52, 58, 53, 51, and 81 in preventative strategies can mitigate lesion severity and reduce LSIL risk. To address this, it’s crucial to give adequate attention to non-16/18 genotypes in screening and vaccination strategies. This study reaffirms that HPV vaccination and cervical cancer screening collectively represent the most effective tools for reducing cervical cancer risks at both individual and population levels. Ongoing research into broader HPV protective vaccines and serotype-specific risks will further inform optimal prevention strategies.

While this study provides valuable insights into HPV prevalence and genotypes in women with cervical lesions in Fujian Province, it is essential to acknowledge certain limitations that may impact the generalizability of findings. The potential for patient selection bias is acknowledged, as the study primarily focused on symptomatic gynecology patients, possibly limiting its representativeness of the general population. Furthermore, financial constraints resulted in some patients declining testing, potentially introducing bias into the study population. The absence of data on relevant factors such as smoking, socioeconomics, and sexual history represents a notable limitation. These factors are known to influence HPV and cervical lesion risk and their exclusion may limit the comprehensive understanding of the study outcomes. Another limitation is the lack of specific analysis of patients’ annual infection rates. The absence of serial HPV data may lead to an underestimation of persistent infections, restricting insights into the long-term dynamics of HPV infection and its impact on cervical lesions.

Despite these limitations, the study’s large sample size contributes to robust data for analysis, mitigating some of the potential drawbacks. However, researchers and practitioners should exercise caution when interpreting the findings in light of these limitations. Future research in this area should strive to address these limitations by incorporating more diverse participant groups, considering relevant influencing factors, and conducting longitudinal analyses to capture the dynamic nature of HPV infection and its relationship with cervical lesions. This ongoing effort will contribute to a more comprehensive understanding of HPV epidemiology and improve the effectiveness of preventive strategies.

## 5 Conclusion

Our large-scale analysis in Fujian Province highlights HPV 52, 58, 53, 51, and 81 as predominant non-16/18 HR-HPV types. Multiple HPV poses increased LSIL risks, while solitary HPV16/18 elevates HSIL and cancer odds. These findings inform tailored cervical cancer prevention, emphasizing specific HPV impacts on lesion severity. The study underscores HR-HPV’s role, emphasizing the need for region-specific prevention strategies, especially for prevalent non-16/18 types in Asia. It provides crucial insights into regional HPV epidemiology, guiding optimized screening. Ongoing surveillance is vital for adapting preventive strategies in the vaccination era.

## Data availability statement

The raw data supporting the conclusions of this article will be made available by the authors, without undue reservation.

## Ethics statement

The studies involving humans were approved by the Ethics Committee of Fujian Maternity and Child Health Hospital, Affiliated Hospital of Fujian Medical University. The studies were conducted in accordance with the local legislation and institutional requirements. Written informed consent for participation was not required from the participants or the participants' legal guardians/next of kin in accordance with the national legislation and institutional requirements.

## Author contributions

YZ: Formal analysis, Writing – original draft, Writing – review & editing. HL: Formal Analysis, Software, Writing – original draft, Writing – review & editing. QY: Data curation, Writing – original draft. YC: Investigation, Software, Writing – review & editing. ZZ: Visualization, Writing – review & editing. JC: Formal analysis, Writing – review & editing. YS: Writing – review & editing. XZ: Funding acquisition, Resources, Writing – original draft, Writing – review & editing. HY: Investigation, Supervision, Writing – original draft, Writing – review & editing. JS: Writing – original draft, Writing – review & editing.
